# Nucleosome Assembly Protein 1, Nap1, Is Required for the Growth, Development, and Pathogenicity of *Magnaporthe oryzae*

**DOI:** 10.3390/ijms23147662

**Published:** 2022-07-11

**Authors:** Qing Wang, Jing Wang, Pengyun Huang, Zhicheng Huang, Yan Li, Xiaohong Liu, Fucheng Lin, Jianping Lu

**Affiliations:** 1 State Key Laboratory for Managing Biotic and Chemical Threats to the Quality and Safety of Agro-Products, College of Life Sciences, Zhejiang University, Hangzhou 310058, China; m15760206862_1@163.com (Q.W.); wj9311@163.com (J.W.); huangpengyun2012@163.com (P.H.); huangzhicheng1210@163.com (Z.H.); leeysmailbox@163.com (Y.L.); 2 Institute of Biotechnology, Zhejiang University, Hangzhou 310058, China; xhliu@zju.edu.cn (X.L.); fuchenglin@zju.edu.cn (F.L.); 3 State Key Laboratory for Managing Biotic and Chemical Threats to the Quality and Safety of Agro-Products, Institute of Plant Protection and Microbiology, Zhejiang Academy of Agricultural Sciences, Hangzhou 310021, China

**Keywords:** rice blast, virulence, septin ring, cell wall integrity, histone, appressorium

## Abstract

*Magnaporthe oryzae* is the causal agent of rice blast, leading to significant reductions in rice and wheat productivity. Nap1 is a conserved protein in eukaryotes involved in diverse physiological processes, such as nucleosome assembly, histone shuttling between the nucleus and cytoplasm, transcriptional regulation, and the cell cycle. Here, we identified Nap1 and characterized its roles in fungal development and virulence in *M. oryzae*. MoNap1 is involved in aerial hyphal and conidiophore differentiation, sporulation, appressorium formation, plant penetration, and virulence. Δ*Monap1* generated a small, elongated, and malformed appressorium with an abnormally organized septin ring on hydrophobic surfaces. Δ*Monap1* was more sensitive to cell wall integrity stresses but more resistant to microtubule stresses. MoNap1 interacted with histones H_2_A and H_2_B and the B-type cyclin (Cyc1). Moreover, a nuclear export signal (NES) domain is necessary for Nap1’s roles in the regulation of the growth and pathogenicity of *M. oryzae*. In summary, *NAP1* is essential for the growth, appressorium formation, and pathogenicity of *M. oryzae*.

## 1. Introduction

*Magnaporthe oryzae* (syn. *Pyricularia oryzae*)-induced rice blast is a highly destructive rice disease capable of causing massive yield reductions in rice or wheat crops worldwide [1]. At the beginning of infection, a three-celled conidium adheres to the rice surface, germinates under suitable conditions (appropriate temperature and humidity), and forms a germ tube, which subsequently differentiates into a melanized, dome-shaped appressorium stimulated by external hydrophobic signals and hardness [2,3]. During appressorium maturation, the glycogen granules and lipid droplets in conidia are gradually degraded and translocated to appressoria, resulting in the accumulation of a high concentration of glycerol, which generates a turgor pressure of up to 8 MPa [4]. The immense turgor provokes great mechanical pressure in an appressorium and promotes its further development into a penetration peg that quickly develops into invasive hyphae in plant cells. After approximately 5–7 days, the typical necrotic spots of rice blast appear on plant surfaces. At this time, a large number of secondary conidia are released from conidial stalks, and start the next round of the infection cycle [5].

Nap1, nucleosome assembly protein 1, was first identified in HeLa cell extracts due to its function in stimulating nucleosome assembly [6]. Nap1 is conserved in eukaryotes and plays diverse biological roles, such as in yeast, *Xenopus*, *Drosophila* [7], *Arabidopsis thaliana* [8], and soybean [9]. In *Drosophila melanogaster*, the deletion of *NAP1* greatly reduces viability [10]. In mice, Nap1 belongs to a multigene family, and the knockout of the neuron-specific *NAP1-homolog-2* gene is embryo lethal [11]. In plants, Nap1 also belongs to a multigene family, and different Nap1 members in tobacco (*Nicotiana tabacum*) and rice (*Oryza sativa*) have different subcellular localizations and appear to perform specific functions [12].

In the nucleosome assembly, Nap1 directly binds core histones and transfers them to naked DNA [13]. In histones, Nap1 has higher affinity levels for binding H_2_A and H_2_B than H3 and H4 in vitro [14]. In *Saccharomyces cerevisiae*, the deletion of *NAP1* led to defects in nucleosome assembly and altered the expression of approximately 10% of nuclear genes [15]. In addition to the nucleosome assembly, Nap1 is involved in the cell cycle [16], histone shuttling between the nucleus and cytoplasm [17], microtubule dynamics and septin assembly [18]. In *S. cerevisiae*, Nap1 was found to bind B-type cyclin 2 (Clb2) and participate in mitosis by regulating Clb2/p34^CDC28^ kinase activity [19]. The protein kinase Gin4 is specifically activated during mitosis, and the phosphorylation and activation of Gin4 during mitosis depend on Clb2 and Nap1 [16]. In animals and yeasts, the dynamic shuttling of Nap1 and Nap1-like proteins between the cytoplasm and nucleus is important for the cell cycle [20]. In *Drosophila* embryonic cells, Nap1 is localized in the nucleus during the S phase and significantly in the cytoplasm during the G2 phase [21]. Similarly, human Nap1L4 appears in the nucleus only during the S phase [22]. Nap1 contains a series of evolutionarily conserved structural domains and motifs, including a nuclear localization signal (NLS) [21], a nuclear export signal (NES) [7], and an acidic segment at the C-terminus that is functionally dispensable for promoting nucleosome assembly [23]. When a nuclear export sequence (NES) is deleted, yeast Nap1 appears to localize in the nucleus [20]. A crystal structure analysis revealed that the NES sequence is covered by a domain that harbors several casein kinase 2 (CK2) phosphorylation sites. The cell cycle-dependent location of Nap1 proteins seems to depend on its phosphorylation state, which is controlled by CK2 [24]. Moreover, Nap1 may have a direct effect on microtubule dynamics. In particular, *NAP1*-deficient yeast cells are resistant to benomyl, a drug that destabilizes microtubules [19]. These data suggest that Nap1 is certainly a multifunctional protein that performs functions in both the nucleus and the cytoplasm.

The function of Nap1 in *M. oryzae* and other pathogenic fungi is not well understood. In this study, we identified the biological functions of Nap1 in *M. oryzae* through gene knockout and phenotypic analyses. We confirmed that MoNap1 interacted with histones H_2_A and H_2_B and a B-type cyclin (Cyc1). However, MoNap1 was primarily localized in the cytoplasm, and the NES domain of MoNap1 was dispensable for localization but necessary for its roles in growth and virulence. Overall, we found that *NAP1* played indispensable roles in regulating the growth, sporulation, appressorium formation, and pathogenicity of *M. oryzae*.

## 2. Results

### 2.1. Identification and Targeted Gene Deletion of NAP1 in M. oryzae

**Figure 1:**
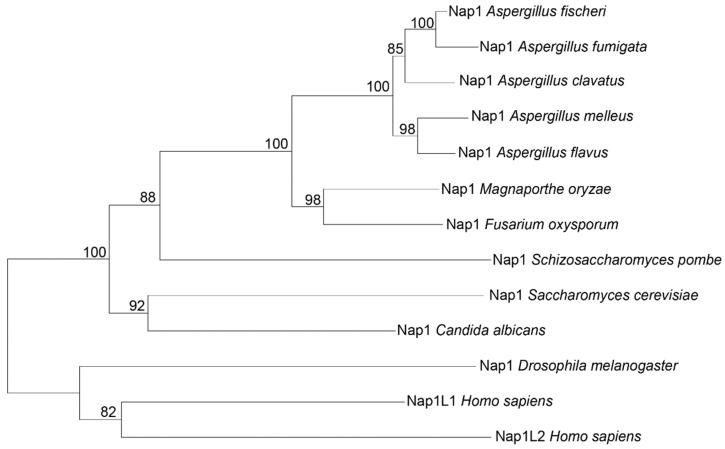
Nap1s are conserved in eukaryotes. Alignment tree of MoNap1 and Nap1 in *S. cerevisiae* (NP_012974.1), *Drosophila melanogaster* (NP_477128.1), *Homo sapiens* (XP_016874829.1, NP_068798.1), *Candida albicans* (XP_718658.1), *Schizosaccharomyces pombe* (NP_587838.1), *Aspergillus melleus* (XP_045950078.1), *Aspergillus clavatus* (XP_001273890.1), *Aspergillus fischeri* (XP_001266074.1), *Aspergillus flavus* (QRD88693.1), *Aspergillus fumigatus* (XP_754077.1), and *Fusarium oxysporum* (XP_031048533.1).

Nucleosome assembly protein 1 in *M. oryzae*, Nap1 (MGG_06924/XP_003709662), was identified by searching for proteins homologous to *S. cerevisiae* Nap1 in the rice blast genome. MoNap1 contains a 404-amino acid polypeptide that shares 45.99% sequence identity with *S. cerevisiae* Nap1 [7]. A phylogenetic tree was constructed based on the amino acid sequences of nucleosome assembly proteins in several species, including *S. cerevisiae* [25], *D. melanogaster* [24], *Homo sapiens* [26], *Candida albicans* [18], *Schizosaccharomyces pombe* [27], *Aspergillus clavatus*, *Aspergillus flavus*, *Aspergillus melleus*, *Neosartorya fischeri*, *Neosartorya fumigate*, and *Fusarium oxysporum*. The alignment tree showed that Nap1 of *M. oryzae* was more homologous to Nap1 of the filamentous fungus *F. oxysporum* than to those of *S. cerevisiae*, C*. albicans*, and *H. sapiens* (Figure 1).

Using the gene replacement strategy, we knocked out *NAP1* in the wild-type *M. oryzae* strain 70-15 (Figure S1A). We verified that the insertion copy number of *HPH* into Δ*Monap1* mutant genome was single by qPCR (Table S1). Based on Δ*Monap1*, we characterized the role of *NAP1* in the growth and pathogenicity of *M. oryzae*. To determine whether the mutant phenotype is caused by the deletion of *MoNAP1*, we built a complementation strain of Δ*Monap1* (*Monap1c*) by transforming the full-length genomic copy of *MoNAP1* into Δ*Monap1*. We also complemented Δ*Monap1* with the *NAP1* gene of *S. cerevisiae* (*Ynap1c*) (Figure S1B–F). In the following section, we describe the biological function of MoNap1 in detail.

### 2.2. NAP1 Is Involved in Sporulation and Appressorium Formation in M. oryzae

**Figure 2:**
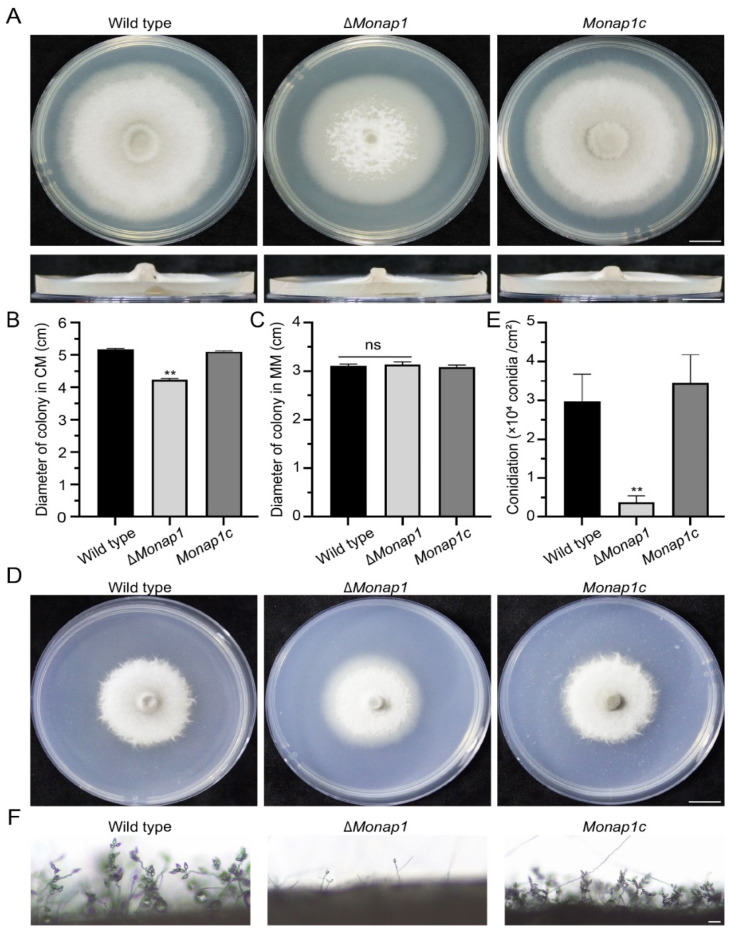
*NAP1* is required for the growth and conidiation of *M. royzae*. (**A**) Colonies of the wild-type, Δ*Monap1*, and *MoNAP1* complemented strains of Δ*Monap1* (*Monap1c*). Bar, 1 cm. (**B**) Mycelial growth (cm) of the wild-type, Δ*Monap1* and *Monap1c* colonies in CM. *n* = 5 independent biological replicates. Error bars represent the standard deviations. The data were analyzed by GraphPad Prism 8.0 and significant differences compared with the wild type were estimated by multiple *t* tests: ** *p* < 0.01. (**C**) Mycelial growth (cm) of the wild type, Δ*Monap1* and *Monap1c* colonies in MM. *n* = 5 independent biological replicates. Error bars represent the standard deviations. The data were analyzed by GraphPad Prism 8.0 and significant differences compared with the wild type were estimated by multiple *t* tests: ns, *p* > 0.05. (**D**) Colonies of the wild type, *ΔMonap1*, and *Monap1c* in MM. Bar, 1 cm. (**E**) Conidiation of the wild type, Δ*Monap1* and *Monap1c* strains in CM. *n* = 5 independent biological replicates. Error bars represent the standard deviations. The data were analyzed by GraphPad Prism 8.0 and significant differences compared with the wild type were estimated by multiple *t* tests: ** *p* < 0.01. (**F**) Conidiophore development of *M. oryzae* strains. Bar, 50 μm.

**Figure 3:**
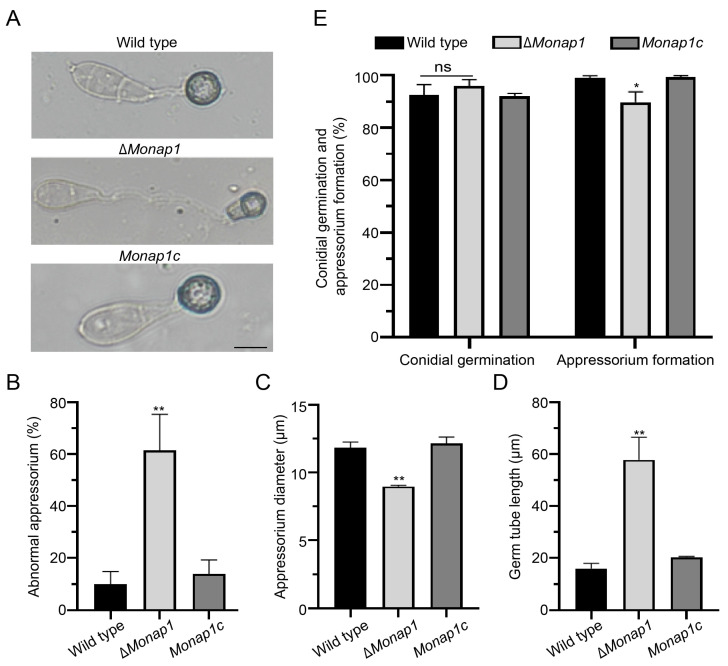
*NAP1* is required for appressorium formation. (**A**) Appressorium formation in the wild-type, Δ*Monap1 an*d *Monap1c* strains on hydrophobic surfaces at 24 hpi. Bar, 10 μm. (**B**) Abnormal appressoria rates (%) in the wild-type, Δ*Monap1* and *Monap1c* strains. At least 150 spores were counted per replicate. *n* = 3 independent biological replicates. Error bars represent the standard deviations. The data were analyzed by GraphPad Prism 8.0 and significant differences compared with the wild type were estimated by multiple *t* tests: ** *p* < 0.01. (**C**) The appressorium diameter in the wild-type, Δ*Monap1* and *Monap1c* strains at 24 hpi. At least 150 spores were counted per replicate. *n* = 3 independent biological replicates. Error bars represent the standard deviations. The data were analyzed by GraphPad Prism 8.0 and significant differences compared with the wild type were estimated by multiple *t* tests: ** *p* < 0.01. (**D**) Germ tube length (μm) in the wild-type, Δ*Monap1* and *Monap1c* strains. Approximately 100 appressoria were photographed and measured using the software NIS-Elements D 3.2 in triplicate. At least 150 spores were counted per replicate. *n* = 3 independent biological replicates. Error bars represent the standard deviations. The data were analyzed by GraphPad Prism 8.0 and significant differences compared with the wild type were estimated by multiple *t* tests: ** *p* < 0.01. (**E**) Conidial germination rate (%) at 4 hpi and appressorium formation rate (%) at 24 hpi in the wild-type, Δ*Monap1* and *Monap1c* strains. At least 150 spores were counted per replicate. *n* = 3 independent biological replicates. Error bars represent the standard deviations. The data were analyzed by GraphPad Prism 8.0 and significant differences compared with the wild type were estimated by multiple *t* tests: ns, *p* > 0.05, * *p* < 0.05.

The Δ*Monap1* mutant grew more slowly than the wild type 70-15 in CM, exhibiting reductions in mycelial growth of approximately 20%. The colony diameter of Δ*Monap1* was 4.24 ± 0.04 cm, while that of the wild type was 5.18 ± 0.02 cm at 9 dpi (days post inoculation) (Figure 2A,B). In addition, the colonies of the mutant appeared thinner and whiter than those of the wild type (Figure 2A). Δ*Monap1* is more susceptible to senescence, which can be observed by the collapse and autolysis of the aerial hyphae at the center of the colony (Figure 2A), indicating that MoNap1 may play a role in the aging process. However, the growth of Δ*Monap1* in MM was comparable to that of the wild type 70-15 (Figure 2C,D). Δ*Monap1* produced significantly fewer spores, approximately 1/8 of the wild type (Figure 2E). In Δ*Monap1*, the mRNA levels of two transcription factor genes *FLBC* and *CON7*, which are involved in the regulation of sporulation, were significantly downregulated (Figure S2). In the wild type, many conidiophores bearing spores in a typical sympodial mode were observed, while Δ*Monap1* produced fewer conidiophores and fewer spores in each conidiophore (Figure 2F). The appressorial morphology of Δ*Monap1* was distorted. The appressoria of the wild type were usually circular or dome-shaped, while approximately 61.5 ± 11.4% of the appressoria in Δ*Monap1* were abnormal (Figure 3A,B) and smaller than those in the wild type (the diameter of appressoria in the wild type was 11.86 ± 0.34 μm, whereas that in the Δ*Monap1* mutant was 8.98 ± 0.07 μm) (Figure 3C). The germ tubes of the Δ*Monap1* mutants were significantly longer than those of the wild type when germinated on hydrophobic plastic coverslips (Figure 3A,D). The germ tube of the wild type was 16.04 ± 1.55 μm in length, whereas that of Δ*Monap1* was 57.89 ± 7.07 μm (Figure 3D). Spore germination was not affected by the *MoNAP1* deletion at 4 hpi (hours post inoculation); however, the appressorial formation rate in Δ*Monap1* was lower than that in the wild type at 24 hpi (89.8 ± 3.2% in Δ*Monap1* compared to 99.0 ± 0.7% in the wild type) (Figure 3E). In addition, turgor pressure of appressoria at 24 hpi was not significantly different between Δ*Monap1* mutants and wild-type (Figure S3).

**Figure 4:**
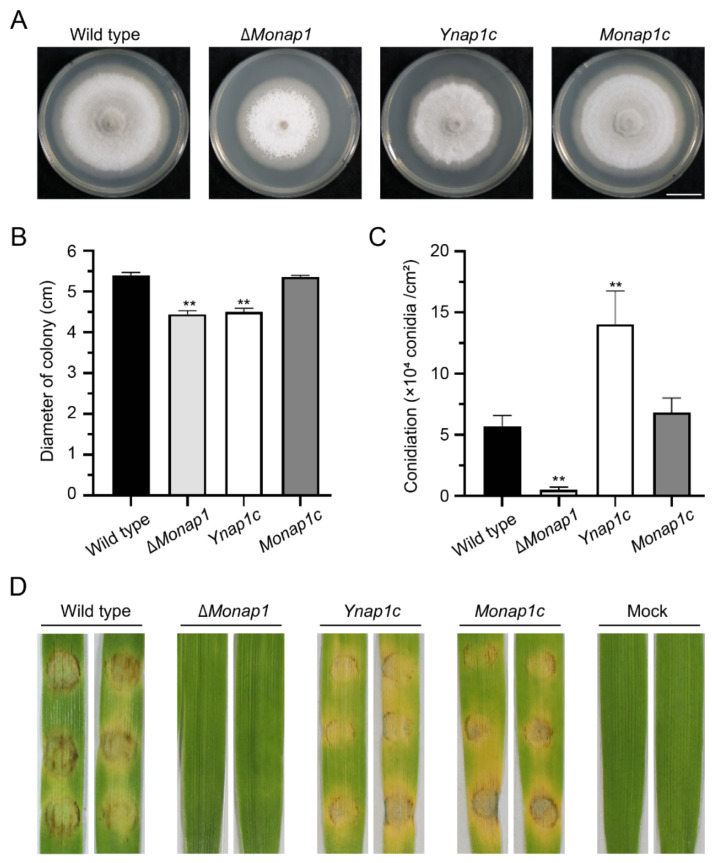
*NAP1* in *S. cerevisiae* can eliminate defects in the sporulation and virulence of Δ*Monap1.* (**A**) Colonies of the wild type, Δ*Monap1*, *NAP1* in *S. cerevisiae* complemented Δ*Monap1* strain (*Ynap1c*) and *Monap1c* strains. Bar, 1 cm. (**B**) Mycelial growth (cm) in the wild type, Δ*Monap1*, *Ynap1c,* and *Monap1c* strains. *n* = 5 independent biological replicates. Error bars represent the standard deviations. The data were analyzed by GraphPad Prism 8.0 and significant differences compared with the wild type were estimated by multiple *t* tests: ** *p* < 0.01. (**C**) Conidiation in the wild type, Δ*Monap1*, *Ynap1c,* and *Monap1c* strains. At least 150 spores were counted per replicate. *n* = 3 independent biological replicates. Error bars represent the standard deviations. The data were analyzed by GraphPad Prism 8.0 and significant differences compared with the wild type were estimated by multiple *t* tests: ** *p* < 0.01. (**D**) Disease symptoms on leaf explants of barley inoculated with mycelial plugs from wild type, ∆*Monap1*, *Ynap1c*, and *Monap1c*. The pictures were photographed at 4 dpi.

The complementation strain *Monap1c* recovered the mutant’s defects in mycelial growth, sporulation, conidiophore formation, and conidium differentiation, indicating that the defects in growth and conidiation of Δ*Monap1* were caused by the deletion of *NAP1*. We also complemented Δ*Monap1* with the *NAP1* gene of *S. cerevisiae* and found that *NAP1* in *S. cerevisiae* can restore the sporulation and virulence of the Δ*Monap1* mutant but not the growth of Δ*Monap1* (Figure 4A–D).

### 2.3. NAP1 Is Required for Virulence in M. oryzae

**Figure 5:**
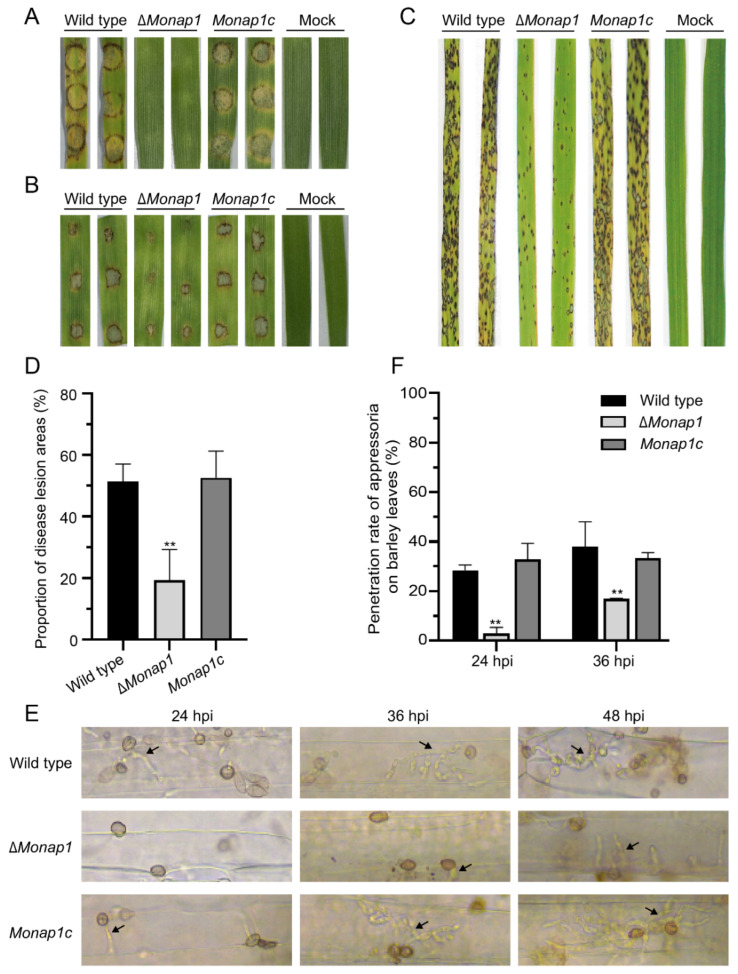
*NAP1* is required for virulence. (**A**) Disease symptoms of leaf explants of barley inoculated with mycelial plugs from the wild-type, ∆*Monap1* and *Monap1c* strains. The pictures were photographed at 4 dpi. (**B**) Disease symptoms of leaf explants of barley inoculated with spore suspensions (5 × 10^4^ conidia mL^−1^). (**C**) Two-week-old rice seedlings were inoculated by spraying 5 × 10^4^ conidia mL^−1^ conidial suspensions from the wild type, ∆*Monap1* and the complemented strain. Lesion severity on rice leaves was evaluated at 7 dpi. (**D**) The proportion of disease lesion areas (%) caused by the wild type, ∆*Monap1*, and *Monap1c* strains on rice seedlings. The area occupied by disease spots per 5 cm of rice leaves was counted. At least 20 leaves were counted per replicate. *n* = 3 independent biological replicates. Error bars represent the standard deviations. The data were analyzed by GraphPad Prism 8.0 and significant differences compared with the wild type were estimated by multiple *t* tests: ** *p* < 0.01. (**E**) Invasive growth of *M. oryzae.* Barley leaf explants were inoculated with 20 μL of conidial suspension (5 × 10^4^ conidia mL^−1^) and cultured for 24 h, 36 h, and 48 h. The arrows indicate invasive hyphae. (**F**) Penetration rate (%) of the wild type, ∆*Monap1* and *Monap1c* appressoria on barley leaves. At least 150 appressoria were counted per replicate. *n* = 3 independent biological replicates. Error bars represent the standard deviations. The data were analyzed by GraphPad Prism 8.0 and significant differences compared with the wild type were estimated by multiple *t* tests: ** *p* < 0.01.

The Δ*Monap1* mutant displayed reduced virulence on barley leaves and rice seedlings. We evaluated the fungal pathogenicity using three inoculation methods. At 4 dpi, the mycelial plugs of the wild-type 70-15 and the complementation strain *Monap1c* caused yellow and brown blast lesions and rotten plant tissues, while the mycelial plugs of ∆*Monap1* did not (Figure 5A). When inoculated on detached barley leaves with 20 μL spore suspensions (5 × 10^4^ conidia mL^−1^) for 4 days, the disease lesions caused by ∆*Monap1* were apparently weaker than those caused by the wild type and the complementation strain (Figure 5B). When sprayed on 2-week-old rice seedlings (CO-39) with conidial suspensions of 5 × 10^4^ conidia mL^−1^, the ∆*Monap1* mutant caused small and restricted lesions in contrast to the typical spindle-like, gray centered, and merged blast lesions caused by the wild type and complementation strain at 7 dpi (Figure 5C). The lesion areas caused by ∆*Monap1* were significantly lower than those caused by the wild type and *Monap1c* (those caused by ∆*Monap1* were 19 ± 8.1%, while those caused by the wild type and *Monap1c* were 52 ± 4.6% and 53 ± 7.0%, respectively) (Figure 5D). Furthermore, we measured the penetration rate of the wild type, ∆*Monap1*, and *Monap1c* at three time points (24 hpi, 36 hpi, and 48 hpi) (Figure 5E). At 24 hpi, 28.4 ± 1.7% and 32.9 ± 5.3% of appressoria penetrated into barley cuticle cells and formed invasive hyphae (IH) structures with the wild type and complementation strain *Monap1c*. However, only 3.1 ± 1.8% of ∆*Monap1* appressoria formed IH structures. At 36 hpi and 48 hpi, the appressorial penetration rate of ∆*Monap1* was still lower than that of the wild type and the complementation strain *Monap1c* (Figure 5F). Moreover, the complemented Δ*Monap1* with the *NAP1* gene of *S. cerevisiae* can restore the virulence of the Δ*Monap1* mutant (Figure 4D).

### 2.4. ∆Monap1 Is Sensitive to Cell Wall Integrity Stresses and Resistant to Microtubule Stresses

**Figure 6:**
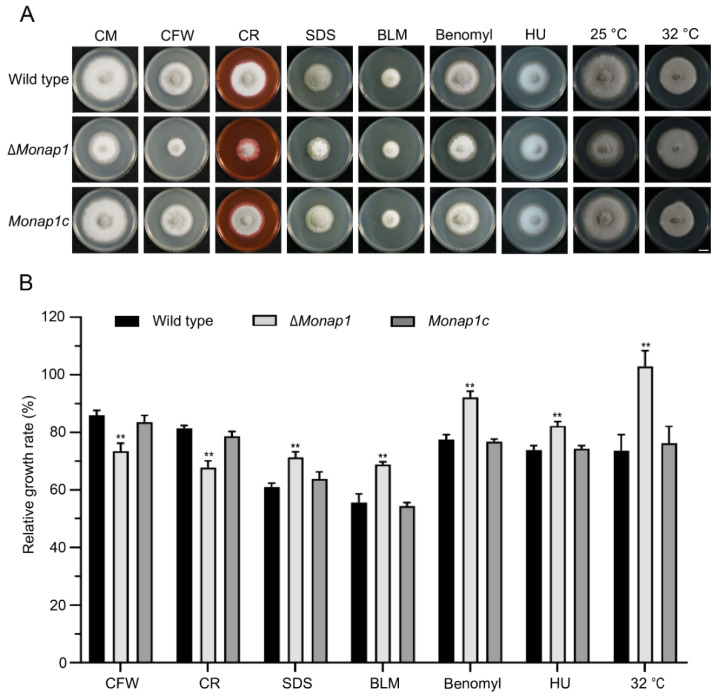
*MoNAP1* is involved in the cell wall integrity (CWI) pathway and microtubule dynamics. (**A**) Mycelial colonies of the wild type, ∆*Monap1* and *Monap1c* strains cultured in CM containing 75 μg mL^−1^ CFW, 600 μg mL^−1^ CR, 0.004% SDS, 35 μg mL^−1^ BLM, 15 μg mL^−1^ benomyl, and 2 mM HU in darkness at 25 °C for 9 days. For the temperature-sensitivity assay, the wild type, ∆*Monap1* and *Monap1c* strains were cultured in CM at 25 °C or 32 °C under a 16 h light and 8 h dark cycle for 9 d. (**B**) Relative growth rate (%) of mycelial colonies in 75 μg mL^−1^ CFW, 600 μg mL^−1^ CR, 0.004% SDS and 35 μg mL^−1^ BLM, 15 μg mL^−1^ benomyl, 2 mM HU and 32 °C growing conditions. *n* = 5 independent biological replicates. Error bars represent the standard deviations. The data were analyzed by GraphPad Prism 8.0 and significant differences compared with the wild type were estimated by multiple *t* tests: ** *p* < 0.01.

The integrity of the cell wall, which is the first barrier contacting the external environment, plays a crucial role in the homeostasis of *M. oryzae* [28]. To assess the role of *MoNAP1* in cell wall integrity stress, the growth of the wild-type 70-15, ∆*Monap1* and *Monap1c* strains was measured in CM with three cell wall integrity stresses, including 75 μg mL^−1^ calcofluor white (CFW), 600 μg mL^−1^ Congo red (CR), and 0.004% sodium dodecyl sulfate (SDS) (Figure 6A). The relative growth rate of mycelial growth of ∆*Monap1* was significantly decreased compared to that of the wild type on CFW and CR (Figure 6B). In contrast, in the presence of 0.004% SDS, ∆*Monap1* showed an increased relative growth rate compared to that of the wild type. In addition, ∆*Monap1* was more resistant to bleomycin (BLM) than the wild type (Figure 6A,B).

In *S. cerevisiae*, the ∆*nap1* mutant showed increased resistance to benomyl, a microtubule destabilizing drug that causes mitotic arrest at high concentrations, suggesting that Nap1 plays a role in regulating microtubule dynamics [19]. Similar to yeast, the ∆*Monap1* mutant grew faster than the wild type in plates containing 15 μg mL^−1^ benomyl in *M. oryzae* (Figure 6A,B). In addition to benomyl, ∆*Monap1* was more resistant to high temperature. ∆*Monap1* exhibited a lower growth defect than the wild type at 32 °C (Figure 6A,B). When wild-type conidia are treated with HU, a DNA replication inhibitor, appressorium formation is blocked [29]. We inoculated wild-type 70-15, ∆*Monap1* and *Monap1c* in CM containing 2 mM HU and found that ∆*Monap1* had a higher relative growth rate than the wild type and *Monap1c* (Figure 6A,B).

### 2.5. Nap1 Is Necessary for Proper Septin Ring Organization in M. oryzae

**Figure 7:**
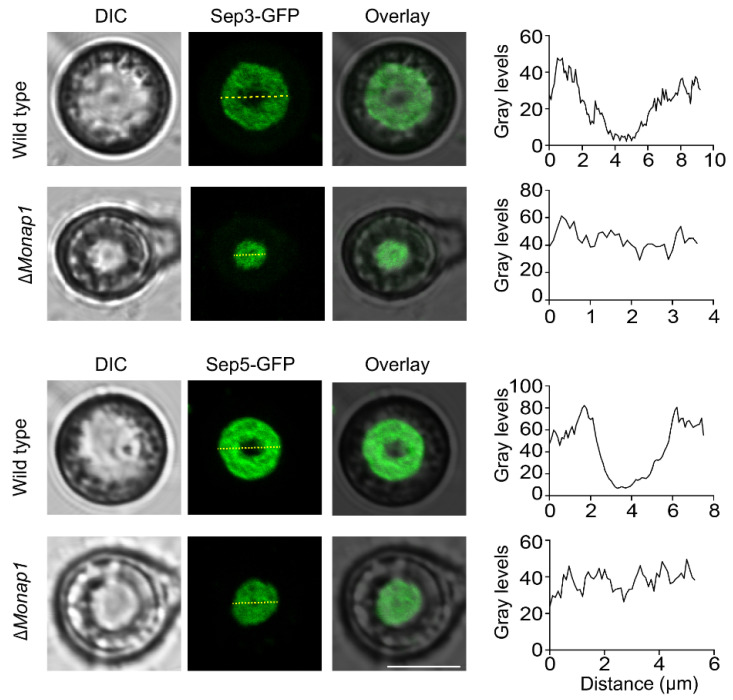
∆*Monap1* exhibits impaired septin organization. Expression of Sep3-GFP (upper panel) and Sep5-GFP (lower panel) in appressoria of *M. oryzae* in the wild type and ∆*Monap1* mutant at 24 hpi. The septin ring showed aberrant distribution and organization in ∆*Monap1*. The distribution of the fluorescence signal in a transverse section (indicated by the yellow line) was analyzed by ImageJ software. Bar, 5 μm.

A critical requirement for appressorium morphology and function in *M. oryzae* is the recruitment and organization of septin-dependent cytoskeletal components [30]. In *C. albicans*, Nap1 plays a role in septin ring organization, and the deletion of *NAP1* affects the stability of the septin ring [18]. The abnormal appressorial morphology of ∆*Monap1* prompted us to observe the localization of the septin ring in the appressorium. We visualized the septin ring by expressing Sep3-GFP and Sep5-GFP fusion proteins in the wild type and mutants. In the wild type, Sep3-GFP and Sep5-GFP showed a ring structure around the appressorium pore, while in the ∆*Monap1* mutant, Sep3-GFP and Sep5-GFP were evenly distributed and appeared similar to a round pie at the bottom of the appressorium at 24 hpi (Figure 7).

### 2.6. Nap1 Binds Histones H_2_A and H_2_B and G2/Mitotic-Specific B-Type Cyclin Cyc1 in M. oryzae

**Figure 8:**
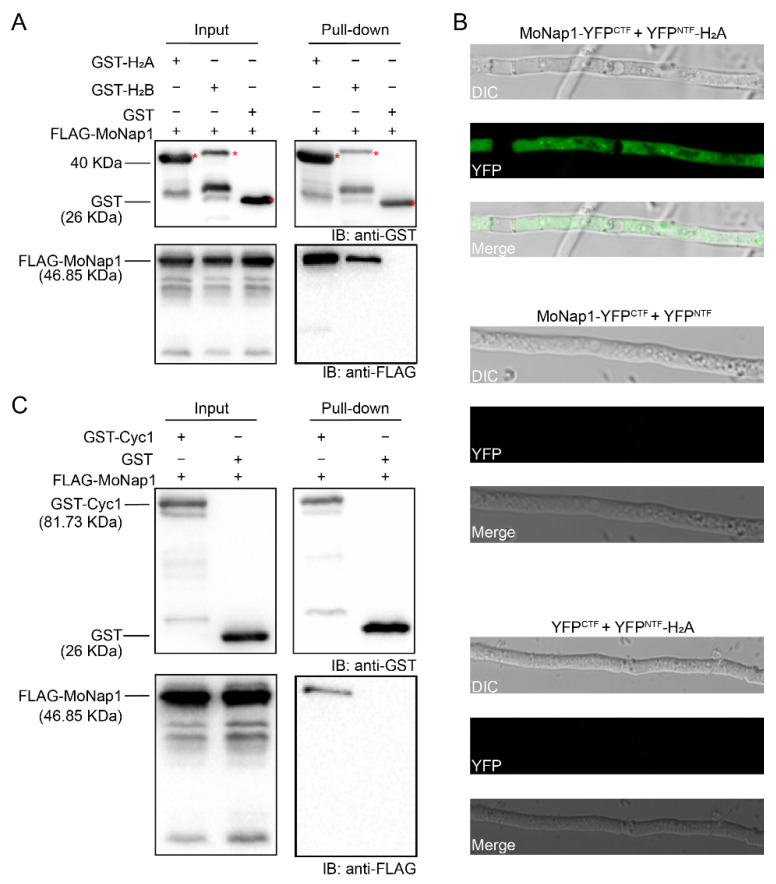
MoNap1 interacted with H_2_A, H_2_B, and Cyc1. (**A**) Pull-down results between FLAG-MoNap1 and GST-H_2_A or GST-H_2_B in *E. coli* BL21. FLAG-MoNap1 was detected in GST-H_2_A and GST-H_2_B eluents but not in GST eluents. Asterisks represent the bands of GST-H_2_A (41.27 kDa) or GST-H_2_B (42.82 kDa). (**B**) Visualization of the MoNap1-H_2_A interaction using a BiFC assay. YFP signals were observed in vegetative hyphae in the transformant harboring MoNap1-YFP^CTF^ and YFP^NTF^-H_2_A. No detectable YFP signals were observed in the negative control transformants harboring MoNAP1-YFP^CTF^ and YFP^NTF^ or YFP^CTF^ and YFP^NTF^-H_2_A. (**C**) Pull-down results between FLAG-MoNap1 and GST-Cyc1. FLAG-MoNap1 was detected in the GST-Cyc1 eluent but not in the GST eluent.

As a nucleosome assembly protein, Nap1 can tightly bind histones H_2_A and H_2_B in yeast [31], *Drosophila* [21], and tobacco [32]. To characterize its biochemical properties in *M. oryzae*, we detected whether MoNap1 bound core histones. Using an in vitro GST pull-down assay, we identified the interaction between MoNap1 and histones H_2_A and H_2_B. The recombinant proteins GST-H_2_A and GST-H_2_B pulled down FLAG-MoNap1, but GST did not (Figure 8A). Furthermore, we detected in vivo protein interactions between MoNap1 and H_2_A using a bimolecular fluorescence complementation (BiFC) assay. MoNap1 was fused to the C-terminal fragment of YFP (YFP^CTF^), and H_2_A was fused to the N-terminal fragment of YFP (YFP^NTF^). The two vectors expressing MoNap1-YFP^CTF^ and YFP^NTF^-H_2_A were cotransformed into the wild type; as negative controls, MoNap1-YFP^CTF^ and YFP^NTF^ as well as YFP^CTF^ and YFP^NTF^-H_2_A were cotransformed into the wild type. Only transformants coexpressing MoNap1-YFP^CTF^ and YFP^NTF^-H_2_A showed YFP signals in the cytoplasm, but the transformants coexpressing two pairs of control vectors did not (Figure 8B).

In budding yeast, Nap1 acts with Clb2 to perform mitotic functions and suppress polar bud growth [19]. In *M. oryzae*, a B-type cyclin gene, *CYC1*, is the homolog of *CLB2* [33]. The different sensitivities of ∆*MoNap1* to benomyl and temperature prompted us to investigate the relationship between Nap1 and the Cyc1 protein. In the pull-down experiments, MoNap1 was pulled down by Cyc1 (Figure 8C). Thus, MoNap1 physically interacted with Cyc1 in *M. oryzae*.

### 2.7. The NES of MoNap1 Is Dispensable for Localization and Binding the Histone Core Proteins H_2_A and H_2_B but Required for Growth and Pathogenicity

**Figure 9:**
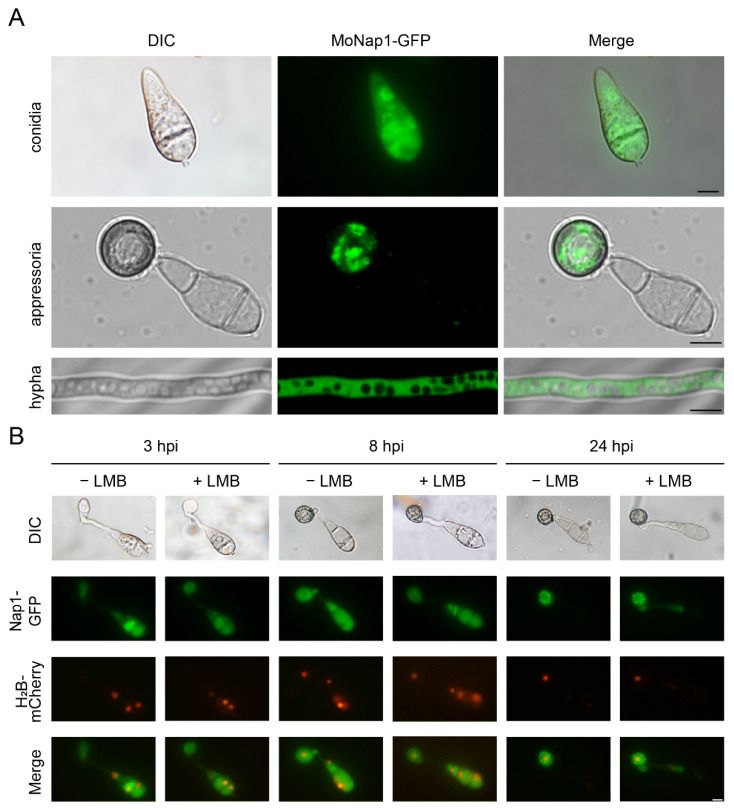
Subcellular localization of MoNap1 in *M. oryzae*. (**A**) Fluorescence signals of MoNap1-GFP in conidial, appressorial and hyphal cells. Bar, 5 μm. (**B**) Fluorescence signals of MoNap1-GFP and H_2_B-mCherry under LMB treatment in conidia and appressoria. Bar, 5 μm.

In both yeast and *Drosophila*, Nap1 has been reported to be a nucleocytoplasmic shuttling protein. However, in *M. oryzae*, MoNap1 mainly localized in the cytoplasm of conidial, appressorial, and hyphal cells (Figure 9A). Additionally, the treatment with leptomycin B (LMB, a specific nuclear export inhibitor) [34] did not result in the obviously increased accumulation of Nap1-GFP in the nucleus (Figure 9B).

**Figure 10:**
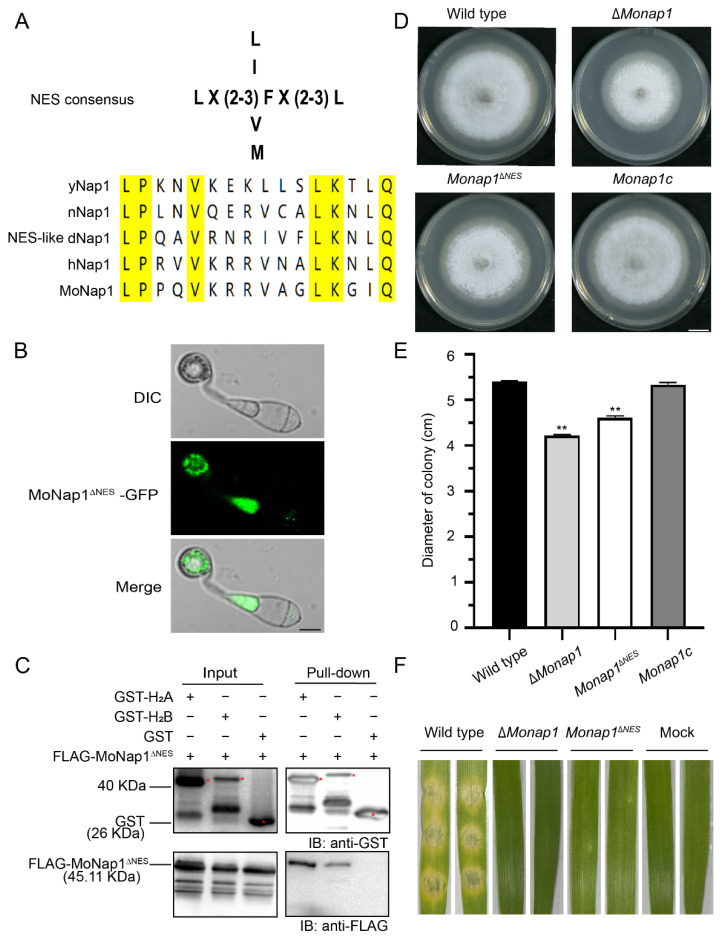
The NES region is not involved in H_2_A/H_2_B binding but is indispensable for growth and pathogenicity. (**A**) NES-like sequences of NAP1. The NES-like sequences of yeast Nap1 (yNap1), nematode Nap1 (nNap1), *Drosophila* Nap1 (dNap1), human Nap1 (hNap1) and MoNap1 are aligned. Yellow highlights indicate conserved amino acids. (**B**) Fluorescence signals of MoNap1^∆NES^-GFP in appressoria. Bar, 5 μm. (**C**) Pull-down results between FLAG-MoNap1^∆NES^ and GST-H_2_A and GST-H_2_B in *E. coli* BL21. FLAG-MoNap1^∆NES^ was detected in GST-H_2_A and GST-H_2_B eluents but not in GST eluents. Red asterisks represent the bands of GST-H_2_A (41.27 kDa), GST-H_2_B (41.82 kDa), and GST (26 kDa). (**D**) Colonies of the wild type and *Monap1*^∆*NES*^. Bar, 1 cm. (**E**) Mycelial growth (cm) of the wild type and *Monap1*^∆*NES*^ colonies. *n* = 5 independent biological replicates. Error bars represent the standard deviations. The data were analyzed by GraphPad Prism 8.0 and significant differences compared with the wild type were estimated by multiple *t* tests: ** *p* < 0.01. (**F**) Disease symptoms of leaf explants of barley inoculated with mycelial plugs from the wild-type and *Monap1^∆NES^* strains. The pictures were photographed at 4 dpi.

In yeast, Nap1 shuttles via a leucine-rich NES [20], and this short peptide sequence is conserved in many species, including nematode Nap1 (nNap1), *Drosophila* Nap1 (dNap1), and human Nap1 (hNap1). MoNap1 contains a 16-amino acid sequence in the N-terminal region that is similar to the NES of the yeast Nap1 protein (Figure 10A). To investigate whether these sequences function as active NES signals, we constructed mutated Nap1^∆NES^ in which Nap1 lacked the corresponding NES region. However, the deletion of the NES sequence in MoNap1-GFP (MoNap1^∆NES^-GFP) did not make Nap1 preferentially localize in the nucleus (Figure 10B).

We examined the possible relationship between histone binding and the NES sequence of Nap1. Therefore, we performed GST pull-down assays with FLAG-MoNap1^∆NES^ and core histones. We found that MoNap1^∆NES^ bound core histones similar to MoNap1 (Figure 10C). Therefore, the NES region is not involved in H_2_A/H_2_B binding.

Nevertheless, we found that *Monap1*^∆*NES*^ exhibited a phenotype similar to that of ∆*Monap1*, including reduced growth and virulence (Figure 10D–F).

## 3. Discussion

Nap1 is a well-conserved protein in eukaryotes, such as mammals, *Drosophila,* and yeast; however, its roles in filamentous fungi are not well understood. *M. oryzae*, an important plant pathogenic fungus, is often used as a model organism in investigating pathogenic fungal-host molecular interactions. In this study, we characterized the biological roles of Nap1 in *M. oryzae* by knocking out *NAP1* and found that Nap1 plays diversified roles in aerial hyphal development, conidiophore and spore differentiation, appressorium formation, and virulence. *NAP1* in *S. cerevisiae* can eliminate the defects in the sporulation and virulence of the Δ*Monap1* mutant, suggesting that Nap1s in *M. oryzae* and *S. cerevisiae* are homologous in both biological function and protein sequence.

In *M. oryzae*, ∆*Monap1* displayed reduced spore production, abnormal appressoria, and decreased infection growth and virulence. The reduced conidiation in Δ*Monap1* is due to its fewer conidiophores and fewer spore numbers on each conidiophore. In *M. oryzae*, several transcription factor genes (*COS1*, *CONX2*, *MSTU1*, *GTA1*, *GCC1*, *FLBC*, *HOX2*, and *CON7*) [35,36,37] and autophagy genes, such as *ATG5* [38] and *TEA4* [39], are involved in the regulation of sporulation. The mRNA levels of two genes, *FLBC* and *CON7*, were significantly downregulated, suggesting a possible pathway regulating conidiation via *FLBC* and *CON7*. In addition, melanin promotes sporulation in the wild type 70-15 [40]. The lower melanin contents in the aerial hyphae of Δ*Monap1* are also responsible for its reduced spore production. *M. oryzae* penetrates the rice cell wall by a melanized, dome-shaped appressorium with a large turgor. In ∆*Monap1*, the appressorium morphology is abnormal and smaller than that in the wild type. Normal appressorium function depends on the microtubule arrangement and F-actin polymerization organized by septin [41]. The septin ring determines the polarity of the appressorium, which is required for plant penetration [42]. Septin GTPases can regulate the organization and functions of microtubule [43]. Moreover, in mammals, the stability of septin disks is dependent on intact microtubules [44]. In *C. albicans*, Nap1 is associated with the assembly of septin rings, and *NAP1* deletion leads to changes in septin dynamics [18]. In tobacco, Nap1 colocalizes with the mitotic spindle and the phragmoplast [12] and interacts with tubulins [32]. Benomyl, a microtubule inhibitor, destabilized microtubules in cells [45]. In *M. oryzae*, the deletion of *MoNAP1* led to increased resistance to benomyl and an increased proportion of abnormal appressoria, suggesting its function in microtubule arrangement. Abnormal cytoskeleton and appressorium morphology cause an abnormal septin ring [30,46]. ∆*Monap1* showed an abnormal distribution of Sep3 and Sep5 at the bottom of the appressorium at 24 hpi. This finding is consistent with the aberrant appressorium morphology of ∆*Monap1*. Thus, the reduced virulence of ∆*Monap1* may be the result of an abnormal appressorium morphology, which is probably related to the altered microtubule dynamics.

A dramatic change in microtubule dynamics occurs when cells enter mitosis. Spindle orientation is a microtubule-dependent process during mitosis that determines the division plane. Mitosis is induced specifically by kinase complexes that contain B-type cyclins and a cyclin-dependent kinase [19]. In yeast, Nap1 interacts with the B-type cyclin Clb2, which is involved in the assembly of the mitotic spindle. Nap1 is required for the ability of Clb2 to suppress polarized bud growth. In *M. oryzae*, Nap1 interacts with the B-type cyclin Cyc1. The deletion of *NAP1* alters the effect of benomyl on microtubule stability and alters microtubule stability during mitosis. In budding yeast, when the wild-type cells were cultured in media containing 11 μg mL^−1^ benomyl, the cells were unable to assemble a functional mitotic spindle. However, ∆*nap1* cells do not exhibit a benomyl-induced mitotic delay [19]. This finding indicates that the deletion of *NAP1* either increases microtubule stability or reduces the requirements for microtubule function during mitosis. In *M. oryzae*, when conidia are treated with HU, a DNA replication inhibitor, the cells are arrested at the S phase, and appressorium formation is blocked [29,33]. We found that ∆*Monap1* also had an increased resistance to HU. Temperature is one of the key factors affecting division of cells, and high temperature inhibits the cell cycle in *Chlamydomonas reinhardtii* [47]. In *A. nidulans*, the *NimA* gene encodes a protein kinase necessary for mitosis [48]. An equivalent gene mutant *MonimA^E37G^* in *M. oryzae* grew normally at 26 °C but showed a reversible growth defect at 32 °C [49]. However, ∆*Monap1* exhibited a higher relative growth rate at 32 °C. Therefore, it is possible that MoNap1 is involved in the regulation of mitosis in *M. oryzae*.

Chitin [(1-4)-β-linked N-acetylglucosamine] is a major structural cell wall component in ascomycetes, and most chitin is fully acetylated and associated with (1-3)-β-glucan and (1-6)-β-glucan [50,51]. In this study, ∆*Monap1* was sensitive to two chitin-binding anionic dyes, CFW and CR, which inhibited the assembly of enzymes that connect chitin to (1-3)-β-glucan and (1-6)-β-glucan [52], indicating that the loss of *MoNAP1* affects the CWI pathway. The CWI pathway controls the cellular remodeling process in response to environmental challenges in fungi [28,52]. Bleomycin (BLM) is a DNA-damaging agent that acts by not only inducing DNA strand breaks [53] but also destroying cell wall components through oxidation and preventing the formation of fungal septum and cytokinesis [54,55,56]. The *nrp1-1 nrp2-1* mutant plants were significantly more sensitive to the bleomycin treatment and increased levels of DNA damage than the wild-type plants in *Arabidopsis* [57]. Conversely, our results showed that ∆*Monap1* increased resistance to BLM. The difference in the response to BLM may be due to the differences in nucleus and cell wall components or the differences in action sites of BLM between *Arabidopsis* and *M. oryzae*.

The localization of Nap1 in the cell has been an interesting issue, and Nap1 exhibits nucleocytoplasmic shuttling in many species. The distinct subcellular localization of Nap1 in the cell reflects functional diversity and complexity. Nap1 is a nucleocytoplasmic shuttling protein in yeast that is primarily localized in the cytoplasm at the steady state but localized in the nucleus when a leucine-rich nuclear export sequence (NES) is mutated [20]. Nap1 binds histone H_2_A/H_2_B dimers in the cytoplasm and participates in the import of histones into the nucleus [58]. The analysis of Nap1 phosphorylation revealed that Nap1 is phosphorylated at 11 sites in vivo in *S. cerevisiae* [59]. In vitro, Nap1 is a substrate for the phosphorylation of CK2 at three serines. Normal S-phase progression requires the reversible phosphorylation of Nap1, and the phosphorylation of Nap1 by CK2 appears to facilitate its import into the nucleus [59]. However, we did not observe increased nuclear localization of MoNap1 after deleting a putative NES, which is consistent with the observation in *C. albicans*, where the deletion of the NES also did not result in NAP1 accumulation in the nucleus [18]. Interestingly, in the BiFC assay, we found that the fluorescent signal was also mainly concentrated in the cytoplasm, which may be related to the fact that NAP1 binds histones in the cytoplasm to form a complex and participates in histone import into the nucleus [60].

In summary, we characterized the roles of Nap1, nucleosome assembly protein 1, in *M. oryzae* by molecular genetic assays and found that *MoNAP1* is required for fungal development and virulence probably through its involvement in microtubule dynamics.

## 4. Materials and Methods

### 4.1. Strains and Culture Conditions

*Magnaporthe oryzae* wild-type strain 70-15 (a strain artificially crossed in the laboratory) and the Δ*Monap1* mutant, the complementary strain of Δ*Monap1* (*Monap1c*), were cultured in complete medium (CM) or minimal medium (MM) at 25 °C under a 16 h light and 8 h dark cycle [61]. For the stress tests, the strains were cultured in CM supplemented with 0.004% sodium dodecyl sulfate (SDS), 75 μg mL^−1^ calcofluor white (CFW), 600 μg mL^−1^ Congo red (CR), 35 μg mL^−1^ bleomycin (BLM), 15 μg mL^−1^ benomyl, or 2 mM hydroxyurea (HU) at 25 °C in darkness for 9 d. For the temperature-sensitive stresses, the strains were cultured in CM at 32 °C under a 16 h light and 8 h dark cycle for 9 days.

### 4.2. Knockout of NAP1 in M. oryzae

An upstream fragment and a downstream fragment of *NAP1* from *M. oryzae* genomic DNA were amplified with the primers Up-F/Up-R and Down-F/Down-R, respectively. The primers used in this study are listed in Supplementary Table S1. A hygromycin B resistance gene, *HPH*, was cloned using the primers HPH-F and HPH-R. Three DNA fragments were fused into a knockout cassette in *Hin*dIII- and *Xba*I-digested pKO3B [62] using a fusion enzyme (Vazyme, Nanjing, China). Then, the knockout cassette was transformed into the wild-type strain via *Agrobacterium tumefaciens*-mediated transformation (ATMT), and the null mutants were confirmed using a previously reported method [36,62]. First, the transformants were screened in selective CM containing 0.5 μM 5-fluoro-2′-deoxyuridine (F2dU) and 200 µg mL^−1^ hygromycin B. Second, the genomic DNA of the transformants was isolated using an improved CTAB method. Then, the null mutants were identified using double PCR in which *MoNAP1* was amplified using the primer set S-F/S-R with *β-TUBULIN* (primers: Tbl-gF/Tbl-gR) as a positive control. The successful recombination of *HPH* into the deleted *MoNAP1* site was confirmed by another PCR using the primer set L-F/HPH-CKR. Third, the insertion copy number of *HPH* in the mutant genome was determined by quantitative real-time PCR (qPCR) using *β-TUBULIN* as a control (primer sets qHPH-F/qHPH-R and qtub-F/qtub-R).

For the complementary strain *Monap1c*, a 3.68 kb native full *NAP1* gene containing a promoter, coding sequence (CDS), and a terminator was cloned from *M. oryzae* wild-type genomic DNA by the primer set NAP1c-F/NAP1c-R and inserted into the *Eco*RI and *Xba*I-linearized vector pKD3, which contains a bialaphos resistance gene (*BAR*) [63]. The complemented DNA fragment was transformed into ∆*Monap1* via ATMT, and the transformants were selected in selection medium containing 750 μg mL^−1^ glufosinate ammonium. The expression of *MoNAP1* in the complementation strains was confirmed at the mRNA level by reverse transcription polymerase chain reaction (RT-PCR). To build a mutated *MoNAP1* in which the NES sequence was deleted (*MoNAP1*^∆*NES*^), two segments (NAP1^1−430 bp^ and NAP1^478−1549 bp^) with primers (NAP1^∆NES^-F1/NAP1^∆NES^-R1 and NAP1^∆NES^-F2/NAP1^∆NES^-R2) were amplified in the genomic DNA of *M. oryzae*, fused to the *Bam*HI-*Xba*I site of pKD3, and then transformed into ∆*Monap1* via ATMT. To complement the *NAP1* of *S. cerevisiae* into *M. oryzae*, a 2.35 kb *NAP1* gene was cloned from *S. cerevisiae* genomic DNA using the primer set yNAP1-F/yNAP1-R and inserted into the *Bam*HI and *Xba*I-linearized vector pKD5, which contains a sulfonylurea resistance gene (*SUR*) [63]. The resulting construct was transformed into ∆*Monap1* by ATMT.

### 4.3. Phenotypic Characterization

The mutant phenotype was assayed according to previous reports [64,65]. To compare mycelial growth, a 5-mm agar block of 8-day-old wild type, ∆*Monap1*, and complementation strain *Monap1c* were inoculated in 7-cm plates containing 17.5 mL CM and cultured at 25 °C with a 16 h light and 8 h dark phase. The colony diameter was measured, and the colonies were photographed 9 days post-inoculation (dpi). Regarding sporulation, conidia were collected from 9-cm plates containing 30 mL CM at 12 dpi and counted with a counting chamber. Regarding conidiophore development, media containing vegetative hyphae were sliced into slender pieces and recultured under continuous light at 25 °C for 24 h [65]. To measure conidial germination and appressorium formation, 25 μL of spore suspension (1 × 10^5^ conidia mL^−1^) were dropped on plastic coverslips and incubated at 22 °C under dark conditions. Conidial germination and appressorium formation were observed at 4 hpi and 24 hpi, respectively.

For the mycelial virulence assays, 5-mm mycelial pellets were inoculated on excised leaves of 7-day-old barley (*Hordeum vulgare*) for 4 days. The virulence assays of rice seedlings (*Oryza sativa cultivar CO39*) were performed by spraying spore suspensions (5 × 10^4^ conidia mL^−1^) in 0.2% (*w/v*) gelatin on 14-day-old rice seedlings. The rice seedlings were cultured in a wet container under dark conditions at 22 °C for 48 h and then recultured under a 16 h light and 8 h dark cycle at 25 °C for 3 days. To assess the disease lesion severity (disease score), the area of a 5-cm-long leaf with the most serious disease lesions in each seedling was counted [65]. Regarding host penetration, 20 μL of spore suspensions (5 × 10^4^ conidia mL^−1^) of the wild-type, ∆*Monap1*, and *Monap1c* strains were dropped onto 7-day-old barley leaves and cultured at 25 °C. At 24 hpi, 36 hpi, and 48 hpi, the leaves were collected, decolored by methanol, fixed in alcoholic lactophenol, and observed under a microscope [64].

### 4.4. Observation of Fluorescence Fusion Proteins in M. oryzae

The fluorescence fusion protein Nap1-GFP was constructed by cloning a 1.5-kb *NAP1* via PCR with a primer set (NAP1-GFP-F/NAP1-GFP-R) from the wild-type genomic DNA and fused with GFP in the *Bam*HI and *Sma*I sites of pKD3-GFP [46]. To express Nap1^∆NES^ -GFP, two segments (*NAP1*^1−430 bp^ and *NAP1*^478−1549 bp^) of the *NAP1* coding sequence were amplified from the genome with primers (NAP1^∆NES^ -GFP-F1/NAP1^∆NES^ -GFP-R1 and NAP1^∆NES^ -GFP-F2/NAP1^∆NES^ -GFP-R2) and fused together with GFP in the pKD3 vector digested with *Xba*I. The fluorescent fusion protein expression vector was transformed into ∆*Monap1* via ATMT. The fluorescence signals were observed under a laser scanning confocal microscope (FV3000). To observe the effects of LMB on the localization of Nap1, 20 nM LMB were added to the conidial suspension, and the fluorescence signals were observed at 3 hpi, 8 hpi, and 24 hpi. To localize Sep3 and Sep5 in the wild type and ∆*Monap1* mutant, the vectors pKD5-Sep3-GFP and pKD5-Sep5-GFP into which *SEP3* or *SEP5* was inserted into the pKD5-GFP vector were transformed into the wild type and ∆*Monap1*, respectively [46].

### 4.5. Pull-Down Assays and Bimolecular Fluorescence Complementation (BiFC) Assay

For the pull-down assays, the cDNA fragments of *MoNAP1* and *MoNAP1*^∆NES^ were inserted into pET21 containing a 3 × FLAG tag, and the cDNA fragments of *H_2_A*, *H_2_B*, and *CYC1* were inserted into pGEX-4T containing a GST tag. The expression vectors were transformed into *Escherichia coli* BL21 separately. FLAG-MoNap1 (46.85 kDa), FLAG-MoNap1^∆NES^ (45.11 kDa), GST-H_2_A (41.27 kDa), GST-H_2_B (41.82 kDa), GST-Cyc1 (81.73 kDa), and GST (26 kDa) were expressed by induction using 0.2 M IPTG for 16 h and pulled down by GST beads C600913 (BBI, Shanghai, China). The proteins were separated by a Western blot assay and detected by an anti-GST antibody EM80701 (HUABIO, Hangzhou, China) or an anti-FLAG antibody M1403-2 (HUABIO, Hangzhou, China).

A BiFC analysis was used to observe the protein-protein interactions in vivo in *M. oryzae*. The plasmids used in the BiFC assay were constructed as previously described [66]. In brief, a *TUBULIN* promoter and a C-terminal fragment spanning approximately amino acids 155 to 238 of YFP (YFP^CTF^) were inserted into the vector pKD9-HPH to form the plasmid pKD9-YFP^CTF^-HPH. The plasmid pKD9-YFP^CTF^-HPH was digested with *Bam*HI and *Sma*I and then ligated with the *MoNAP1* fragment amplified with a primer pair (NAP1- YFP^CTF^-F/NAP1- YFP^CTF^-R), resulting in the final pKD9-MoNAP1-YFP^CTF^-HPH plasmid. Another N-terminal fragment spanning approximately amino acids 1 to 154 of YFP (YFP^NTF^) was inserted into the vector pKD5-SUR and a *TUBULIN* promoter to form the plasmid pKD5-YFP^NTF^-SUR. The plasmid pKD5-YFP^NTF^-SUR was digested with *Sal*I and then ligated with the H_2_A fragment amplified with a primer pair (H_2_A-YFP^NTF^-F/H_2_A-YFP^NTF^-R), resulting in the final pKD5-YFP^NTF^-H_2_A-SUR plasmid. Next, the pKD9-MoNAP1-YFP^CTF^-HPH fusion vector was transformed into the 70-15 strain in combination with pKD5-YFP^NTF^-H_2_A-SUR. The transformants were screened in CM containing hygromycin B and sulfonylurea, and at least three independent transformants were examined under an LSM710nlo laser scanning confocal microscope (Zeiss, Jena, Germany).

## Data Availability

All data supporting the findings of the current study are available within figures and supporting information. All strains generated during this study are available from the corresponding author upon reasonable request.

## Supplementary Materials

The following supporting information can be downloaded at: www.mdpi.com/article/10.3390/ijms23147662/s1.
