# Supplementary material for: Nucleosome Assembly Protein 1, Nap1, Is Required for the Growth, Development, and Pathogenicity of Magnaporthe oryzae
Source: Int J Mol Sci. 2022 Jul 11;23(14):7662. doi: 10.3390/ijms23147662 (PMC9316785; doi:10.3390/ijms23147662)
Supplement: Supplementary file 1 [file ijms-23-07662-s001.zip › ijms-1764071-supplementary.pdf]

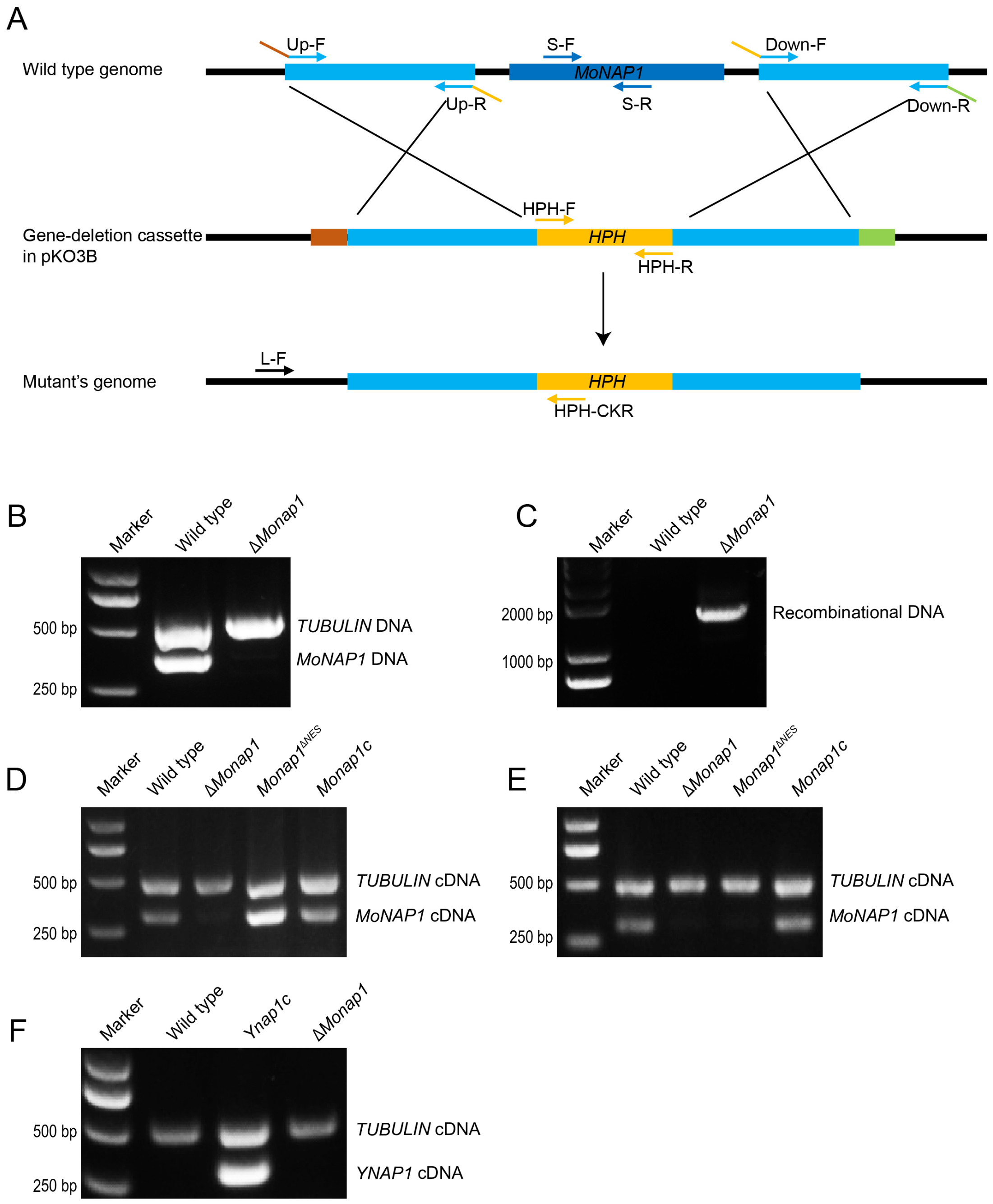

Figure S1. Knockout and complementation of *NAP1* in *Magnaporthe oryzae* strain 70-15. (**a**) Targeted gene deletion strategy. Two pairs of primers (Up-F/Up-R and Down-F/Down-R) were used to clone the respective left and right flanking fragments of the targeted genes. The primer set S-F/S-R was used to clone a fragment of the targeted deletion gene in transformants. The primer set L-F/HPH-CKR was used to clone the recombinational DNA fragments in mutants. (**b**) *MoNAP1* deletion events in wild-type 70-15 were confirmed at the DNA level by double PCRs of both *MoNAP1* (amplified by primers S-F/S-R) and the positive control of *β-TUBULIN* DNA (amplified by primers Tbl-gF/Tbl-gR). (**c**) Recombinational DNA

was confirmed at the DNA level by positive PCR using the primer set L-F/HPH-CKR. (**d**) Complementation of Δ*Monap1* by *MoNAP1* (*Monap1c*). Complemented *MoNAP1* was confirmed at the RNA level by RT–PCR using *β-TUBULIN* as a control. The primer set cNAP1-F/cNAP1-R was used to clone a fragment of the complemented gene in the transformants. (**e**) Complementation of Δ*Monap1* by *MoNAP1* without the NES sequence (*Monap1*$^{\Delta NES}$). The primer set cNAP1$^{\Delta NES}$ -F/cNAP1$^{\Delta NES}$ -R was used to verify the deletion of the NES sequence at the RNA level, where a fragment could be cloned in the wild type, but no fragment was cloned in *Monap1*$^{\Delta NES}$. (**f**) Complementation of Δ*Monap1* with the *NAP1* gene of *S. cerevisiae* (*Ynap1c*). Complemented *YNAP1* was confirmed at the RNA level by RT–PCR using *β-TUBULIN* as a control. The primer set cNAP1y-F/cNAP1y-R was used to clone the *NAP1* gene of *S. cerevisiae.*

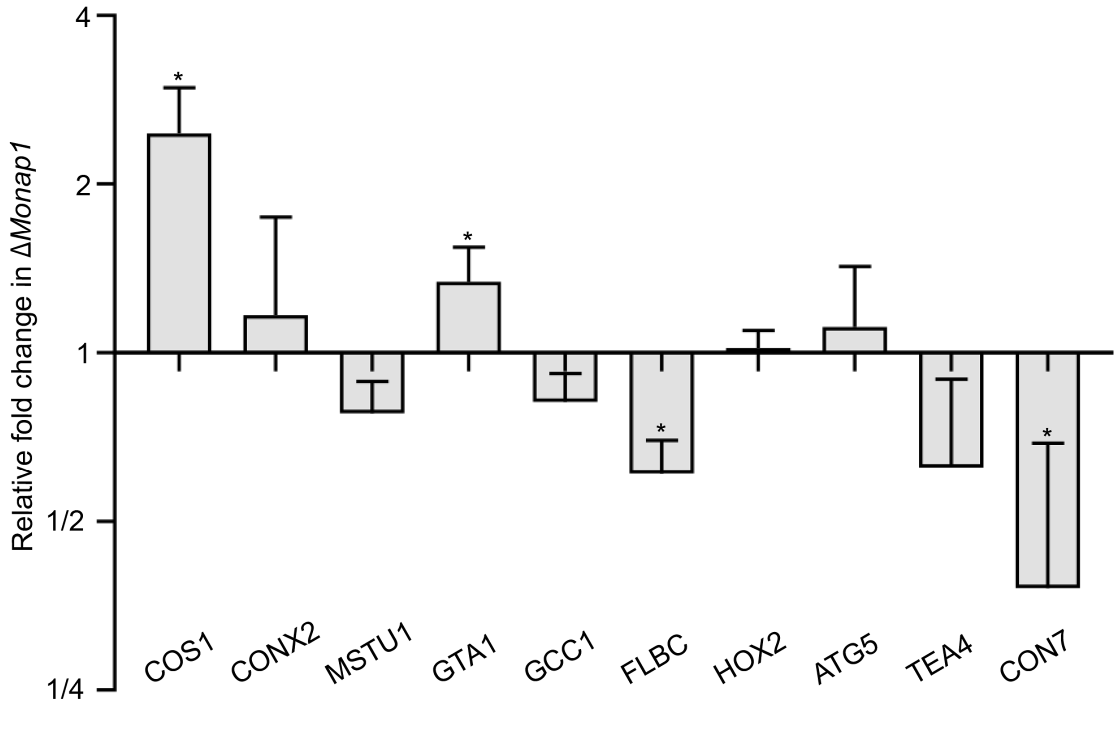

Figure S2. Expression levels of the conidiation-related genes *COS1, CONX2, MSTU1, GTA1, GCC1, FLBC, HOX2*, *CON7*, *TEA4*, and *ATG5* in Δ*Monap1*. Error bars represent the standard deviations. The data were analyzed by GraphPad Prism 8.0 and significant differences compared with the wild type were estimated by multiple t tests: * $p < 0.05$.

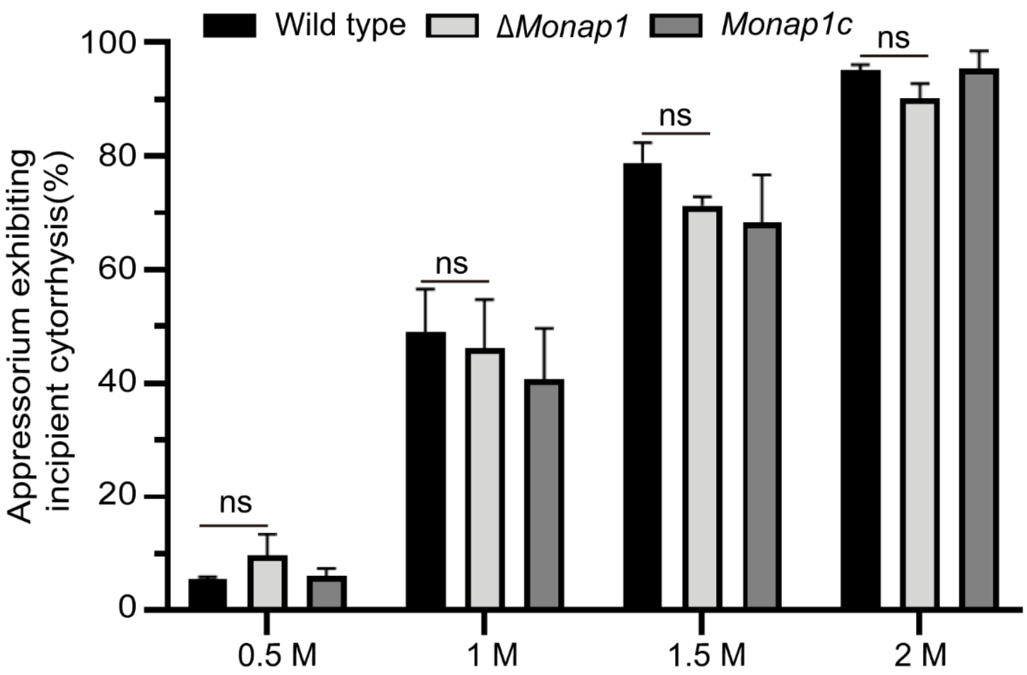

Figure S3. Collapsed appressoria rates (%) of the wild-type, Δ*Monap1*, and *MoNAP1* complemented strains of Δ*Monap1* (*Monap1c*). At least 150 spores were counted per replicate. $n = 3$ independent biological replicates. Error bars represent the standard deviations. The data were analyzed by GraphPad Prism 8.0 and significant differences compared with the wild type were estimated by multiple t tests: ns, $p > 0.05$.

Table S1. Copy number identification of the selectable marker gene *HPH* inserted in the genome of Δ*Monap1*.

Gene	Gene locus (MG8)	Parent strain	Recombination resistance gene	Knockout[a]	Copies of *HPH* by qPCR[b]	Insertion event[c]
MoNAP1	MGG_06924	70-15	*HPH*	Y	0.99	Single

Notes:

a "Y" and "N" represent the target gene deleted or undeleted, respectively.

b The copy number of the selectable marker gene (*HPH*) in the genome of the mutants identified by qPCR after normalized with β-tubulin gene and the wild type strain.

c "Single" represents the targeted gene deletion without ectopic insertion.

Table S2. Primers used in this study

1 Primers used to build gene deletion cassettes

Up-F	GCCCGGGAGATGGGGGAGGCTAACTGACACTCTAGATACTCGAGGTCTACC
Up-R	AAAATAGGCATTCATTGTTGACCTCCACTA TGCTGCATGGCGTGATT
Down-F	CCCAGCACTCGTCCGAGGGCAAAGGAATAGGTGACGGTGCTAGTCTGTTT
Down-R	TCACGACGTTGTAAAACGACGGCCAGTGCCAAGCTTGGTTTGCTCAGTCCATTGTTATG
HPH-F	TAGTGGAGGTCAACAATGAATG
HPH-R	CATCTACTCTATTCCTTTGCCC

2 Primers used to identify Δ*Monap1*, *Monap1*c, *Monap1*$^{\Delta NES}$ and *Ynap1c*

S-F	CATCGTCATCGTCCTCATCTT
S-R	GAGCAGAAGAAACAGCGAAAC
L-F	AGACAGCAATTCCATGACG
HPH-CKR	GGCTGATCTGACCAGTTGCCT
Tbl-gF	TTCCGCGCTGTCACCGTTCC
Tbl-gR	GGGCCTCCTCCTCGTACTCCTCTT
cNAP1-F	GAAGAAGGAGGTCAAGACTGAAG
cNAP1-R	CGGCATGGTCGTAGATGAA
qtub-F	ATTGTTCACCTTCAGACCGG
qtub-R	TTGAAGTAGACGCTCATACGC
qHPH-F	ATGTCCTGCGGGTAAATAGC
qHPH-R	GATGCAATAGGTCAGGCTCTC
cNAP1$^{\Delta NES}$ -F	AAGTGAAGCGCCGAGTGGCTG
cNAP1$^{\Delta NES}$ -R	GGCCAGGGAAATTTGGTTCTTC
cNAP1y-F	CGCAAAGGGCCAAGAGATTG
cNAP1y-R	TGGGTTGGCGGAAGAATCAA

3 Primers used in the complementation experiments

NAP1c-F	TCACCGAGATTTAGGAATTCCCCAGCGACAAACTCAAA
NAP1c-R	TACTGCAGGTCGACTCTAGAACAGAATACTGCAGGATCAAAC
NAP1$^{\Delta NES}$ -F1	TCAATCACAATGGCCGGATCCATGGCCGAGCCCATTCCC
NAP1$^{\Delta NES}$ -R1	TCAAGTTTGGAGTGCTCCTTTGACTCGATGTAGCCAGAAG
NAP1$^{\Delta NES}$ -F2	AAGGAGCACTCCAAACTTGA
NAP1$^{\Delta NES}$ -R2	TACTGCAGGTCGACTCTAGACTAGCTCTGCTTGCACTCGG
yNAP1-F	TCAATCACAATGGCCGGATCCATGTCAGACCCTATCAGAACGAAAC
yNAP1-R	TTACTGCAGGTCGACTCTAGAGGTGGCATTGCCCAAGTCC

4 Primers used to construct NAP1-GFP and NAP1$^{\Delta NES}$ -GFP plasmid

NAP1-GFP-F	CAATCACAATGGCC GGATCC ATGGCCGAGCCCATTCCC
NAP1-GFP-R	CCCTTGCTCACCATCCCGGGGCTCTGCTTGCACTCGGC
NAP1$^{\Delta NES}$ -GFP-F1	ACGAGCTGTACAAGTCTAGAATGGCCGAGCCCATTCCC
NAP1$^{\Delta NES}$ -GFP-R1	TCAAGTTTGGAGTGCTCCTTTGACTCGATGTAGCCAGAAG
NAP1$^{\Delta NES}$ -GFP-F2	AAGGAGCACTCCAAACTTGA

NAP1$^{\Delta NES}$ -GFP-R2	TACTGCAGGTCGACTCTAGACTAGCTCTGCTTGCACTCGG
**5 Primers used in BiFC and pull-down assays**	
NAP1- YFP^{CTF}-F	CAAACCATAAAAATGGGATCCATGGCCGAGCCCATTCCC
NAP1- YFP^{CTF}-R	CTTGCAGGCCGGGCGCCCGGGGCTCTGCTTGCACTCGGCG
H_2A-YFP^{NTF}-F	AGAAAGGTATCTAGAGTCGACATGACTGGAGGCGGCAAGTCCGG
H_2A-YFP^{NTF}-R	TGTCGCTTACTGCAGGTCGACCATCTCTTGGCTGGCGTTCTT
FLAG-NAP1-F	ATGATGACGACAAGGTCGACATGGCCGAGCCCATTCCCAA
FLAG-NAP1-R	TCGAGTGCGGCCGCAAGCTTGCTCTGCTTGCACTCGGCGG
GST-H_2A-F	CGCGTGGATCCCCGGAATTCATGACTGGAGGCGGCAAGTC
GST-H_2A-R	TCGAGTCGACCCGGGAATTCCATCTCTTGGCTGGCGTTCT
GST-H_2B-F	CGCGTGGATCCCCGGAATTCATGCCCCCCAAGGCCGCTGA
GST-H_2B-R	TCGAGTCGACCCGGGAATTCTTTGGTGCTTGAAGAGTACT
GST-CYC1-F	CGCGTGGATCCCCGGAATTCCCCCCAGCCCGGACTGCGC
GST-CYC1-R	TCGAGTCGACCCGGGAATTCCAGCTGGTCGATAGCTACG
**6 Primers used in qPCR**	
COS1-qF	CACAACCATTCAAAGACGCAC
COS1-qR	AGAGTTGACTTTGGCGATGAG
CONX2-qF	GCCCGAGAGCAAGAGTAATAC
CONX2-qR	CTGCTTCATGTACGGGTAGTC
MSTU1-qF	GATCAGGGTCACAATAACGGG
MSTU1-qR	ACTAGAGATGGACGAGACAGG
GTA1-qF	AAGACATCCACGACCAGTTC
GTA1-qR	GTAACGTGGTTCCTGGTAGC
GCC1-qF	CCAAGTTTGAAAGCTCCGAG
GCC1-qR	GTGAACAAAGGGCAAGGAAG
FLBC-qF	TCCCTCAACTACGACCCTTAC
FLBC-qR	GTGAGCTGCCAGAGTAGATAC
HOX2-qF	GGACTGCCTCTCAATGGTATG
HOX2-qR	AGGTGTGGGAGCAATTATCG
ATG5-qF	GGGTCTTTTAGGGTCATGCAG
ATG5-qR	AGCAAGTCACGTAGTGTTGG
TEA4-qF	AGCACATTGAGACACCCAC
TEA4-qR	GGCTACAGTCTTGGTCTTCC
CON7-qF	ACCATTTAAACGCGCATGTG
CON7-qR	CGTTCCTCGTCTGCCTTG
